# Supplementary material for: Experimental Induction of Intracranial Aneurysms in Rats: A New Model Utilizing a Genetic Modification within the EDNRA Gene
Source: Brain Sci. 2022 Sep 14;12(9):1239. doi: 10.3390/brainsci12091239 (PMC9497172; doi:10.3390/brainsci12091239)
Supplement: Supplementary file 1 [file brainsci-12-01239-s001.zip › brainsci-1906715-supplementary.pdf]

Supplementary Materials

Experimental Induction of Intracranial Aneurysms in Rats: A New Model Utilizing a Genetic Modification within the EDNRA Gene

Tim Lampmann[1,*], Valeri Borger[1], Jürgen Konczalla[2], Suzana Gispert[3], Georg Auburger[3], Hartmut Vatter[1] and Erdem Güresir[1]

[1] Department of Neurosurgery, University Hospital Bonn, 53127 Bonn, Germany
[2] Department of Neurosurgery, University Hospital Frankfurt, 60528 Frankfurt am Main, Germany
[3] Experimental Neurology, Medical Faculty, Goethe University, 60590 Frankfurt am Main, Germany

*Corresponding author: tim.lampmann@ukbonn.de

1. *Supplementary Results*

1.1. Non-invasive blood pressure measurement

BP was normally distributed for all groups at all time points except for EDNRA + HT at week 8 and WT_CTL at week 12, as assessed by the Shapiro-Wilk test ($\alpha = 0.05$).

Homogeneity of variances could be assumed for all time points (week 4: $p = 0.166$; week 8: $p = 0.392$; week 10: $p = 0.186$; week 12: $p = 0.102$).

BP was normally distributed for WT_CTL at week 4, week 8 and week 10, but not for week 12, as assessed by the Shapiro-Wilk test ($\alpha = 0.05$). Homoscedasticity could be assumed, $\chi^2(5) = 3.26$, $p = 0.661$. There was no statistically significant difference for the time points, $F(3, 36) = 0.55$, $p = 0.651$.

BP was normally distributed for EDNRA_CTL at all time points as assessed by the Shapiro-Wilk test ($\alpha = 0.05$). Homoscedasticity could be assumed, $\chi^2(5) = 8.37$, $p = 0.138$. There was no statistically significant difference for the time points, $F(3, 36) = 2.75$, $p = 0.057$.

BP was normally distributed for WT + HT at all time points as assessed by the Shapiro-Wilk test ($\alpha = 0.05$). Homoscedasticity could be assumed, $\chi^2(5) = 6.95$, $p = 0.233$. There was statistically significant difference for the time points, $F(3, 18) = 7.96$, $p = 0.001$.

BP was normally distributed for EDNRA + HT at week 4, week 10 and week 12, but not for week 8, as assessed by the Shapiro-Wilk test ($\alpha = 0.05$). Homoscedasticity could be assumed, $\chi^2(5) = 2.66$, $p = 0.755$. There was no statistically significant difference for the time points, $F(3, 24) = 1.28$, $p = 0.306$.

1.2. Immunofluorescence

MGV was normally distributed for EDNRA + HT, but not for WT_CTL and EDNRA_CTL, as assessed by the Shapiro-Wilk test ($\alpha = 0.05$).

Homogeneity of variances could not be assumed for MGV ($p = 0.002$).

1.3. Functional investigation

E_{max} was normally distributed for all groups as assessed by the Shapiro-Wilk test ($\alpha = 0.05$).

pD_2 was normally distributed for WT_CTL, WT + HT and EDNRA + HT, but not for EDNRA_CTL, as assessed by the Shapiro-Wilk test ($\alpha = 0.05$).

Homogeneity of variances could be assumed for E_{max} and pD_2 ($p = 0.615$ and $p = 0.365$, respectively).

2. *Supplementary Tables*

Table S1. Non-invasive blood pressure measurement: Means and SD of BP

Time point	Group	Animals [N]	Mean [mmHg]	SD
**Week 4**	**WT_CTL**	13	127.23	12.08
	**EDNRA_CTL**	13	136.92	10.95
	**WT + HT**	12	185.33	20.86
	**EDNRA + HT**	17	203.00	17.93
**Week 8**	**WT_CTL**	13	165.62	36.36
	**EDNRA_CTL**	13	126.69	16.90
	**WT + HT**	11	131.23	11.51
	**EDNRA + HT**	17	167.27	24.63
**Week 10**	**WT_CTL**	13	209.29	21.12
	**EDNRA_CTL**	13	162.06	40.06
	**WT + HT**	10	127.38	19.23
	**EDNRA + HT**	17	131.77	12.29
**Week 12**	**WT_CTL**	13	166.20	23.49
	**EDNRA_CTL**	13	204.76	25.86
	**WT + HT**	7	160.6	39.35
	**EDNRA + HT**	9	122.92	10.20

N=number of samples; SD=standard deviation

Table S2. Immunofluorescence: Means and SD of MGV

Group	Animals [N]	Images [N]	Mean	SD
**WT_CTL**	9	32	8.63	3.65
**EDNRA_CTL**	8	27	8.49	4.90
**WT + HT**	3	21	9.45	2.06
**EDNRA + HT**	6	54	9.46	2.82

N=number of samples; SD=standard deviation

Table S3. Functional investigation: Means and SD of E_{max}

Group	Measurements [N]	Mean	SD
**WT_CTL**	9	85.12	17.24
**EDNRA_CTL**	19	86.15	15.95
**WT + HT**	11	101.75	23.84
**EDNRA + HT**	11	75.40	19.92

N=number of samples; SD=standard deviation

Table S4. Functional investigation: Means and SD of pD_2

Group	Measurements [N]	Mean	SD
**WT_CTL**	9	7.79	0.27
**EDNRA_CTL**	19	7.54	0.22
**WT + HT**	11	7.78	0.33
**EDNRA + HT**	11	7.80	0.23

N=number of samples; SD=standard deviation

Table S5. Functional investigation: Means and SD of single data points

Group	ET-1 concentration [M]	Measurements [N]	Mean	SD
**WT_CTL**	**10^{-11}**	9	-1.91	2.02
	**10^{-10}**	9	-2.09	1.93
	**10^{-9}**	9	0.30	3.34
	**3×10^{-9}**	9	6.44	9.27
	**10^{-8}**	9	33.68	13.04
	**3×10^{-8}**	9	49.64	11.57
	**10^{-7}**	9	85.12	17.24
	**3×10^{-7}**	9	58.16	28.77
**EDNRA_CTL**	**10^{-11}**	19	-1.43	4.24
	**10^{-10}**	19	-1.77	3.64
	**10^{-9}**	19	1.52	4.31
	**3×10^{-9}**	19	5.08	6.93
	**10^{-8}**	19	23.31	13.65
	**3×10^{-8}**	19	37.95	17.34
	**10^{-7}**	19	86.15	15.95
	**3×10^{-7}**	19	50.78	21.84
**WT + HT**	**10^{-11}**	11	1.69	1.87
	**10^{-10}**	11	1.02	0.92
	**10^{-9}**	11	2.10	5.47
	**3×10^{-9}**	11	5.91	7.48
	**10^{-8}**	11	39.52	22.35
	**3×10^{-8}**	11	59.31	21.87
	**10^{-7}**	11	101.75	23.84
	**3×10^{-7}**	11	88.46	28.80
**EDNRA + HT**	**10^{-11}**	11	3.36	4.58
	**10^{-10}**	11	4.48	3.69
	**10^{-9}**	11	7.22	10.13
	**3×10^{-9}**	11	7.30	11.19
	**10^{-8}**	11	28.40	13.02
	**3×10^{-8}**	11	50.65	11.60
	**10^{-7}**	11	73.21	23.79
	**3×10^{-7}**	11	52.66	20.09

N=number of samples; SD=standard deviation
